# Whole-exome sequencing of 228 patients with sporadic Parkinson’s disease

**DOI:** 10.1038/srep41188

**Published:** 2017-01-24

**Authors:** Cynthia Sandor, Frantisek Honti, Wilfried Haerty, Konrad Szewczyk-Krolikowski, Paul Tomlinson, Sam Evetts, Stephanie Millin, Thomas Keane, Shane A. McCarthy, Richard Durbin, Kevin Talbot, Michele Hu, Caleb Webber, Chris P. Ponting, Richard Wade-Martins

**Affiliations:** 1 Oxford Parkinson’s Disease Centre, University of Oxford, Oxford, United Kingdom; 2 MRC Functional Genomics Unit, Department of Physiology, Anatomy and Genetics, University of Oxford, Oxford, OX1 3PT, United Kingdom; 3 Nuffield Department of Clinical Neurosciences, John Radcliffe Hospital, West Wing Level 6, Headley Way, Oxford OX3 9DU, United Kingdom; 4 Wellcome Trust Sanger Institute, Wellcome Trust Genome Campus, Hinxton, Cambridge, CB10 1SA, United Kingdom; 5 Department of Physiology, Anatomy and Genetics, University of Oxford, Oxford, OX1 3QX, United Kingdom

## Abstract

Parkinson’s disease (PD) is the most common neurodegenerative movement disorder, affecting 1% of the population over 65 years characterized clinically by both motor and non-motor symptoms accompanied by the preferential loss of dopamine neurons in the substantia nigra pars compacta. Here, we sequenced the exomes of 244 Parkinson’s patients selected from the Oxford Parkinson’s Disease Centre Discovery Cohort and, after quality control, 228 exomes were available for analyses. The PD patient exomes were compared to 884 control exomes selected from the UK10K datasets. No single non-synonymous (NS) single nucleotide variant (SNV) nor any gene carrying a higher burden of NS SNVs was significantly associated with PD status after multiple-testing correction. However, significant enrichments of genes whose proteins have roles in the extracellular matrix were amongst the top 300 genes with the most significantly associated NS SNVs, while regions associated with PD by a recent Genome Wide Association (GWA) study were enriched in genes containing PD-associated NS SNVs. By examining genes within GWA regions possessing rare PD-associated SNVs, we identified *RAD51B*. The protein-product of *RAD51B* interacts with that of its paralogue *RAD51*, which is associated with congenital mirror movements phenotypes, a phenotype also comorbid with PD.

Parkinson’s disease (PD) is a common, multifactorial and genetically heterogeneous neurodegenerative disorder, primarily of old age. It is characterized by progressive loss of dopamine neurons in the substantia nigra pars compacta together with the accumulation of intracellular protein inclusions termed Lewy bodies^1^. Highly penetrant Mendelian variants, such as in *SNCA, LRRK2, PARKIN, PINK1*, and *PARK7* genes, explain less than 10% of familial PD^2^. Twenty-eight DNA variants across 24 loci that predict risk, albeit to a minor degree, for sporadic PD have been identified using genome-wide association studies (GWAS) involving over ten thousand individuals with PD^3^.

Despite this progress, most sporadic PD cases remain unexplained genetically. With larger studies and the increased application of genome sequencing technologies, it is anticipated that there will be a better understanding of the genetic contributions to PD risk that would directly inform on the molecular and cellular etiology of the disease. Exonic sequence is highly enriched in variants that explain the heritability of complex traits^4^. By sequencing the exons of protein-coding genes (in other words, the exome) of PD subjects, and identifying their variants, which are rare in a control cohort, there is the prospect of identifying further PD disease genes. Nevertheless, only when very large cohorts (>10,000 samples) are investigated will there likely be sufficient power for such individual rare disease-associated variants to be revealed^5^.

Variants that occur more frequently in the patient cohort than in controls could still be of interest for predicting PD susceptibility despite individually not being genome-wide significant. For example, individual genes, or else multiple genes encoding functionally interacting proteins, might be found that harbour an unusually large number of such sub-genome-wide significant variants. These genes would then add to our understanding of the molecular deficiencies contributing to neurodegeneration in PD.

Here, we analyse exonic variants found in 228 individuals with sporadic PD, all recruited from a relatively homogeneous UK population. We apply multiple approaches at the level of the variant, the gene and the pathway to analyse whether – considered individually or together – these variants can contribute to PD diagnosis.

## Results

Exomes of 244 Parkinson’s disease (PD) patients selected from the Oxford Parkinson’s Disease Centre Discovery Cohort were sequenced. After quality control, and after one individual with relatively strong identity-by-descent (>0.1875) was discarded, 228 PD patient exomes were considered for analysis and compared with 884 control exomes from UK10K cohort and matching our control in term of ancestry (Method). 94,369 sites with base calls in at least 99% of samples were retained for analysis. Eight individuals known to carry variants in *GBA* (N370S and L444P) or *LRRK2* (R1441C and G2019S) were included as positive controls for mutation detection through exon resequencing. All known variants were detected, although the single *GBA* L444P variant call failed to exceed the VQSLOD threshold. We called a total of 94,369 single nucleotide variants (SNVs), of which 39,569 had a minor allele frequency of less than 1% (in controls), affecting 10,252 genes. Of 94,369 SNVs, 21,235 were non-synonymous (NS) affecting 9444 protein coding genes, including 313 that caused the gain or loss of a stop codon and 6,541 that were considered by Polyphen-2^6^ as being damaging (Supplementary Fig. 1).

### No SNV/gene reached genome-wide significance for PD association

**Figure 1:**
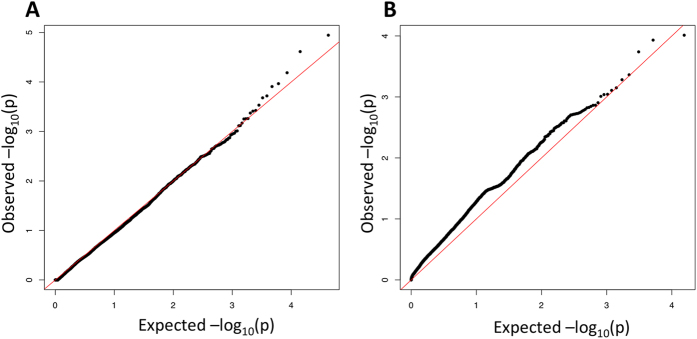
No SNV/gene reached genome-wide significance for PD association. Quantile–quantile plot of p-values of (**A**) logistic regression analyses and (**B**) sequence kernel association test (SKAT) where two PC ancestry were used as covariates and considering only non-synonymous variants.

**Figure 2:**
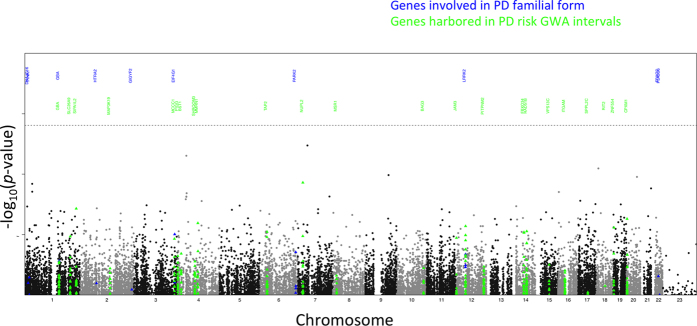
Manhattan plot showing the significance (−log_10_(*p*-value)) of association of non-synonymous variants with PD status. The horizontal line indicates the Bonferroni multiple testing correction threshold at *p* = 2.4 × 10^−6^. Amongst the black points, the blue and green points represent the significance of association for non-synonymous variants within genes involved in the familial form of PD (blue) or genes harboured in one of 26 published PD GWA intervals (green), respectively. The names of genes are given in the top part of plot in blue and green for mono and GWA genes respectively. For 26 GWA intervals, only the name of the gene(s) that possesses the most significant association from the exome non-synonymous variants is showed.

Fisher’s exact test and logistic regression using between 1 and 10 covariates were performed using PLINK^7^ (http://pngu.mgh.harvard.edu/purcell/plink/). The best-fitting quantile-quantile (Q–Q) plot was obtained with the use of two covariates in logistic regression and the control sets listed in the Methods (Figs 1A and 2). As expected, given the low number of cases and the multiple testing correction required, no substitution achieved significance of association with PD status (*p*-value upper threshold 2.4 × 10^−6^; Fig. 2). We next used SKAT^8^ to perform a burden test which found no single gene showing a significant association of multiple SNVs with PD risk. (Fig. 1B and Supplementary Fig. 2). After performing burden analyses, we again failed to find a significant enrichment in the number of NS per individual between cases and controls (NS_cases_ = 6639 vs NS_controls_ = 6650, *p*-value = 0.34).

### PD association in previous reported PD candidate genes

We then examined globally whether known genes involved in the familial or sporadic forms of PD were enriched in PD associated NS SNV (Method). For 15 genes involved in familial PD^9^ (Supplementary Table 1), we did not find a significant enrichment of PD-associated NS SNVs (10/15 genes had at least one NS SNV; *p*-value enrichment based on a logistic regression test = 0.054 and *p*-value enrichment based on the SKAT test = 1). However, for 329 genes harboured within 26 GWA intervals (Methods), a significant 1.2-fold enrichment in PD-associated NS SNVs was achieved (163/329 genes had at least one NS SNV, *p*-value enrichment based on logistic regression test = 0.036).

Next, we looked amongst 15 genes involved in familial and 329 genes harboured in 26 GWA intervals (Fig. 1, Tables 1 and 2) for NS SNVs with nominal p-value associations of <0.05 with PD. We identified two PD associated NS SNVs with MAF < 1% within genes harboured in PD risk GWA intervals, each with an odds ratio >3.5. Specifically, these were (1) rs34094401, a missense mutation (L172W) in the *RAD51B* gene encoding a protein essential for DNA repair (18 cases versus 20 controls are heterozygous for this variant) lying in the PD GWA interval defined by the lead SNP rs1555399, and (2) rs41309351, a missense mutation (R480W) in the *CPXM1* gene encoding for a carboxylpeptidase (16 cases versus 12 controls are heterozygous for the variant) lying in the PD GWA interval defined by the lead SNP rs55785911. Truncating and missense variants in the RAD51B-interacting paralog *RAD51* have been reported to cause congenital mirror movement syndrome^10^,^11^. By examining the frequencies for rs34094401 in an independent cohort including 536 PD patients and 260 controls matching PD patients for age and genotyped on HumanCoreExome chip from Illumina^12^, we observed a similar but non-significant effect at the *RAD51B* locus (*p*-value = 0.14): MAF cases = 0.01306 (14 cases heterozygous) vs MAF controls = 0.005769 (3 controls heterozygous). We noted that the control minor allele frequency of these two SNPS were consistent with those reported by the Exome Aggregation Consortium (**ExAC**) in the European (Non-Finnish) population^13^: rs34094401 MAF_ctr_ = 0.010180995 & MAF_ExAC_ = 0.011834053 while rs41309351 MAF_ctr_ = 0.00678733 & MAF_ExAC_ = 0.003578.

### Functional analyses of PD-associated SNVs

Although we did not find genome-wide significant results for PD association, genes whose proteins contribute to a common cellular process or pathway could contain an enrichment of SNVs that are sub-genome-wide significant for PD association. For this analysis, we examined whether the top *N* genes with most PD-associated NS SNVs (*N* = 100, 200, 300) were enriched in particular Gene Ontology terms^14^. Only when considering the top 300 genes were any significant enrichments found, specifically *extracellular matrix part* (GO:0044420; 1.8-fold enrichment; *q*-value = 0.02) and *extracellular matrix disassembly* (GO:0022617; 1.8-fold enrichment; q-value = 0.02) (Supplementary Table 2). Although only significant when considering such a large number of genes, the genes annotated with a role in *extracellular matrix disassembly* include those encoding the metalloproteinases MMP-7 and MMP-8, and the reduced expression of metalloproteinases has been noted in PD post-mortem brain tissue^15^ while a polymorphism in MMP-9 has been associated with PD and amyotrophic lateral sclerosis^16^.

## Discussion

In this study, we compared the exomes of 228 PD cases with 884 controls exomes drawn from the UK10K^17^ study. We performed association tests both at the level of single-nucleotide variants and at the gene level but found that no variant was significantly associated with PD after applying a multiple-testing correction. While power for discovery is clearly the major issue, there may be additional considerations affecting our study. As the heritability of PD is not high (34–40%), the identification of causative variants or risk alleles for PD may be more difficult than for some other, common neurological disorders^18^,^19^. Furthermore, as our controls are not aged-matched, we do not know whether control individuals will go on to develop PD. Indeed, for example the *LRRK2*-G2019S mutation has a penetrance of 28% at age 59 years, 51% at age 69 years, and 74% at age 79 years^20^. Burden tests or collapsing methods, such as SKAT, might be expected to improve power over single-marker tests, but they still require thousands of samples to reach acceptable statistical power^8^,^21^.

We considered different subsets of the UK10K^17^ controls for this study and the best-fitting Q–Q plot was obtained using a set of six UK10K data sets^17^ (see Methods). Notably for other studies seeking exome controls, the use of the two largest UK10K cohorts, COHORT_TWINS (~1,700 samples) and COHORT_ALSPAC (740 samples), produced inflated p-values that could not be controlled by filtering or covariates. Some of the minor allele frequencies from these two cohorts that were responsible for this effect were very different from the allele frequencies in other controls, suggesting a population stratification problem. While DNA sequencing enables the direct detection of causal mutations and may supersede microarray-based genotyping methods, some of the limitations of GWAS still apply to these studies. Thousands of cases and controls are required to robustly associate rare variants with a common disease and population stratification has to be controlled for^5^,^22^.

NS SNVs most associated with PD were enriched both in genes whose proteins have roles associated with the extracellular matrix and in genes lying within PD-associated GWA intervals. Furthermore, we identified a nominally-associated SNV in the *RAD51B* gene which lies within a region of the genome associated with PD through GWA. Variants in the paralog *RAD51*, whose protein product interacts with that of *RAD51B*, have been associated with congenital mirror movement disorders (OMIM 614508) and mirror movement phenotypes are also associated with PD^23^. Thus, *RAD51B* represents an interesting candidate gene for further examination within this GWA locus.

## Methods

### Clinical characteristics of PD subjects

Established in September 2010, the Oxford Discovery Cohort (www.opdc.ox.ac.uk) comprises patients with idiopathic PD diagnosed in the previous 3.5 years according to UK PD Society Brain Bank diagnostic criteria^24^ recruited from a 2.4 million Thames Valley population with the aim of following up the cohort over the natural history of their disease. PD patients were prospectively recruited over two years from October 2010 to September 2012 and underwent a 2-hour standardised assessment with a movement disorders neurologist and research nurse^25^, following which patients were diagnosed as having PD, with an estimated clinical probability for this diagnosis being made by the research neurologist. Atypical parkinsonian features were systemically screened for using the NINDS Parkinson’s tool, and patients with secondary parkinsonism due to head trauma or medication use, or features of atypical parkinsonism syndromes, were excluded from the study. Subjects were also excluded from subsequent analysis if they had a <80% baseline probability of manifesting idiopathic PD. Clinical assessments were performed while the subject was taking their normal medications, and in the ‘on’ state. The study was undertaken with the understanding and written consent of each subject, with the approval of the NHS South Central Berkshire Research Ethics Committee (study reference 10/H0505/71), and in compliance with national legislation and the Declaration of Helsinki.

244 consecutive PD subjects from the Discovery cohort were recruited to this exome study, of whom 224 (90%) were selected on the following basis: (i) possessing a *LRRK2* R1441C or G2019S variant (n = 4), (ii) possessing a *GBA* N370S or L444P variant (n = 4), (iii) subject had undergone MRI brain scan (n = 20), (iv) subject had undergone Cerebrospinal Fluid (CSF) examination (n = 39), v) subject had undergone skin biopsy (n = 16), (vi) subject had at least 1 first degree PD affected relative (n = 52), (vii) subject had at least 1 second degree PD affective relative (n = 21) and (viii) subject had a Montreal Cognitive Assessment (MoCA) score ≤22 indicating significant cognitive impairment (n = 68). Of the 244 PD subjects, the male to female ratio was 0.60:0.40, mean (SD) age at diagnosis was 66.3 (9.4) years (age range 31.6–87.4 years, mean (SD) Hoehn and Yahr scale was 1.90 (0.5) (range 1.0–4.0, median = 2, interquartile range = 2, 2) and mean (SD) disease duration from diagnosis was 1.7 (1.1) years. Ethnicity of the PD subjects comprised 92% white British, 2.6% white Irish, 2.9% white other, 0.4% Indian, and 2.1% other.

### Control Exome samples

As our PD exome patients have ben recruited in UK, we used public whole-exome sequencing data of individuals matching our PD cohort in term of ancestry and thus with UK origin. The following exome sample datasets were used from the UK 10K dataset: NEURO_IOP_COLLIER (152 samples), NEURO_ABERDEEN (317 samples), NEURO_EDINBURGH (213 samples), RARE_NEUROMUSCULAR (72 samples), RARE_THYROID (18 samples) and NEURO_IMGSAC (112 samples), making a total of 884 control exomes. The two largest UK10K^17^ cohorts, COHORT_TWINS and COHORT_ALSPAC, were not used as they contained minor allele frequencies that differed from those of other control cohorts, indicating a possible population stratification problem. Furthermore, to avoid inflation of low p-values due a stratification issue with particular subsets of the UK10K controls, best-fitting Q–Q plot was obtained using the set of six control data sets.

### Exome sequencing

120 μl of 25 ng/μl of DNA was fragmented to an average size of 150 bp (75–300 bp), and subjected to Illumina DNA sequencing library creation using Bravo automated liquid handling. Adapter ligated libraries were amplified via 6 cycles of PCR. 500 ng/ul of each amplified library was hybridized to RNA baits (SureSelect Human All Exon 50 Mb) and post sequence capture processed following the Agilent SureSelect protocol. Enriched libraries were post capture indexed using a unique DNA barcode via 12 cycles of PCR. Equimolar pools of 8 libraries were sequenced using the Illumina HiSeq machine using the 75 base paired end protocol.

### Data processing

Sequencing reads from each lane were aligned to the human reference genome used in the 1000 Genomes phase 2 (NCBI build 37 + decoy v5)^26^ using BWA version 0.5.9-r16 and the parameters ‘-q 15 –t 6’. The GATK^27^ ‘IndelRealigner’ was used to realign reads near known indels [ ftp://ftp.1000genomes.ebi.ac.uk//vol1/ftp/technical/reference/phase2_mapping_resources/]. The BAM files were then re-sorted and quality scores were recalibrated using GATK ‘TableRecalibration’^27^ ignoring all SNP positions in dbSNP v135^28^. Finally, SAMtools ‘calmd’ was used to recalculate MD/NM tags in the BAM files. All lanes from the same library were then merged into a single BAM file using Picard tools^29^ and PCR duplicates were marked using Picard ‘MarkDuplicates’. Finally, the library BAM files were merged into a single BAM per individual.

### Variant calling

SNP and indel calls were made using samtools^30^ by pooling the alignments from all 246 individual exome BAM files. A genotype likelihood (bcf) file was created with samtools (v0.1.18-r572) mpileup, for every site in the exome bait regions (+/−100bp) [samtools mpileup –EDVSp –C50 –L500 –m3 –F0.2 –d 160000 –P ILLUMINA –l baits.w100.bed]. Variants were then called using bcftools (v0.1.18-r572) [bcftools view –m 0.9 –vcgN].

### SNP filtering

Variant Quality Score Recalibration (VQSR)^31^ was used to filter SNPs. The GATK (v1.6-13-g91f02df) UnifiedGenotyperwas used to recall the sites/alleles discovered by samtools in order to generate annotations to be used for recalibration. The GATK VariantRecalibrator was then used to model the variants, followed by GATK ApplyRecalibration which assigns VQSLOD (variant quality score log odds ratio) values to the variants. Annotations used for training were QD, HaplotypeScore, MQRankSum, ReadPosRankSum, FS, MQ, and InbreedingCoeff. The training sets were HapMap3.3 and Omni2.5M sites. The truth set used was HapMap3.3 sites while the known set was dbSNP 132 sites. A VQSLOD cut off was chosen and the following filters applied to SNP sites:

LowQual,Description = “Low quality variant according to GATK (GATK)”

MinVQSLOD,Description = “Minimum VQSLOD score [SNPs:−1.8356, truth sensitivity 99.5]”.

### Indel filtering

Indels were left-aligned using GATK (v1.6-13-g91f02df) LeftAlignVariants. The following filters were then applied to indel sites:

StrandBias,Description = “Min P-value for strand bias (INFO/PV4) [0.0001]”

BaseQualBias,Description = “Min P-value for baseQ bias (INFO/PV4) [1e-100]”

EndDistBias,Description = “Min P-value for end distance bias (INFO/PV4) [0.0001]”

GapWin,Description = “Window size for filtering adjacent gaps [3]”

MaxDP,Description = “Maximum read depth (INFO/DP or INFO/DP4) [8000000]”

MinAB,Description = “Minimum number of alternate bases (INFO/DP4) [2]” >  MinDP,Description = “Minimum read depth (INFO/DP or INFO/DP4) [16000]”

MinMQ,Description = “Minimum RMS mapping quality for SNPs (INFO/MQ) [10]”

Qual,Description = “Minimum value of the QUAL field [10]”

RefN,Description = “Reference base is N []”.

### Additional filtering

Additionally, the following filters were applied to individual genotype calls based on their depth or genotype quality:

IndelsMinSampleDP,Description = “Genotypes set to. for samples with DP < 4 (FORMAT/DP)”

IndelsMaxSampleDP,Description = “Genotypes set to. for samples with DP > 2000 (FORMAT/DP)”

SNPsMinSampleGQ,Description = “Genotypes set to. for samples with GQ < 20 (FORMAT/GQ)”

IndelsMinSampleGQ,Description = “Genotypes set to. for samples with GQ < 60 (FORMAT/GQ)”.

### Annotation

The calls were annotated with dbSNP 137 rsIDs and population allele frequencies from the 1000 Genomes Phase 1^26^ integrated (v3) callset [ ftp://ftp.1000genomes.ebi.ac.uk//vol1/ftp/phase1/analysis_results/integrated_call_sets]. Variant consequence annotations were added using the Ensembl Variant Effect Predictor^32^ (v2.4) against Ensembl 66. This provides coding consequence predictions and SIFT, PolyPhen-2^6^ and Condel annotations.

### Association analysis

VCF files were converted to PED format (format of file used by PLINK (Purcell *et al*. 2007)) with VCFtools 87^33^ [—remove-indels –remove-filtered-all]. We performed single variant association analyses by using logistic regression test incorporating two first ancestry covariate. We evaluated evidence for association at the genetic level by using the sequence kernel association test (SKAT)^8^ that allows for risk and protective effects in the same gene”. We used asymptotic p-values and incorporated ancestry covariate (two first Principal Components). SKAT assumes that rare variants are more likely to be causal variants with large effect sizes and assigns weight to the variant according to minor allele frequency (we used the default frequency weight (1 and 25 as the two beta distribution shape parameters, to up-weight lower frequency mutations)). As power was clearly an issue in this study, we did not repeat these analyses by functional category of variants.

### PD GWA intervals

The 26 PD GWA intervals were defined: (i) by identifying the most distant pair of SNPs with either (i) an r^2^ > 0.5 with the lead SNP reported in http://pdgene.org^3^,^34^ using the haplotypes of 1000 Genome Project or (ii) an r^2^ > 0.5 computed by considering the European haplotypes only, and then adding an additional 250 kb on either side of the interval to include genes that may be regulated by GWA-associated regulatory variants.

### Gene Sets enriched in genes with PD-associated Non-synonymous SNVs

To evaluate if a set of genes was enriched in PD associated NS SNVs, we computed the sum of -log_10_ of p-value association of logistic regression test of NS SNVs within these genes and compared this measure with this got with random set genes matching each gene of test gene set for the number of NS SNVs. We performed an analogue test by replacing the p-value association of logistic regression by p-value associated with SKAT test.

#### Tissue enrichment analysis

FPKM=> Feature Scaling=> Euclidean Normalization (such that the square root of the sum of the square of expression levels is one). Compare the sum of expression value for PD risk gene with a set of random gene (match for CDS length).

### Overlap with PD GWAS intervals

26 genome-wide significant PD intervals were acquired from http://pdgene.org 3,34. Intervals were extended at each end by 500kb. The number of the top *N* genes (10 ≤ *N* ≤ 100) most significantly associated by SKAT with PD located within either set of intervals was compared with the number of randomly sampled length-matched genes in these intervals.

## Additional Information

**Accession codes:** Data for this study, EGAS00001000151, is accessible through the European Genome-phenome Archive (https://www.ebi.ac.uk/ega/home).

**How to cite this article**: Sandor, C. *et al*. Whole-exome sequencing of 228 patients with sporadic Parkinson’s disease. *Sci. Rep.* **7**, 41188; doi: 10.1038/srep41188 (2017).

**Publisher's note:** Springer Nature remains neutral with regard to jurisdictional claims in published maps and institutional affiliations.

## Supplementary Material

Supplementary Information

## Figures and Tables

**Table 1:** Suggestive PD risk non-synonymous variant (p-value < 0.05) within genes known to be involved in familial form of PD.

SNV	Chromosome	Position	Gene	AA subtitution	Allele	MAF (Cases)	MAF (Controls)	Odds Ratio	Pval
rs2302464	4	15709252	BST1	R/Q	A/G	0.018	0.039	0.44	0.042
rs1801334	6	161781225	PARK2	D/N	T/C	0.026	0.046	0.56	0.038
rs33995883	12	40740686	LRRK2	N/D	G/A	0.020	0.010	1.96	0.044
rs2270968	3	182755209	MCCC1	H/P	T/G	0.285	0.240	1.26	0.014

**Table 2:** Suggestive PD risk non-synonymous (p-value < 0.05) within genes harbored in PD GWA intervals.

SNV	Chromosome	Position	Gene	AA	Damaging prediction	Allele	MAF (Cases)	MAF (Controls)	Odds Ratio	Pval
rs2228396	6	32797809	TAP2	A/T	PolyPhen = benign(0.06); SIFT = tolerated(0.3); GMAF = T: 0.0983	T/C	0.061	0.097	0.61	0.008
rs61736778	12	40837165	MUC19	T/I	PolyPhen = possibly_damaging(0.74); SIFT = tolerated(0.34); GMAF = T: 0.0115	T/C	0.022	0.011	1.96	0.048
rs2114566	12	40814107	MUC19	I/V	PolyPhen = benign(0); SIFT = tolerated(0.33); GMAF = G: 0.0445	G/A	0.029	0.018	1.64	0.01
rs10506156	12	40823474	MUC19	V/I	PolyPhen = benign(0); SIFT = tolerated(0.7); GMAF = A: 0.0450	A/G	0.029	0.018	1.64	0.016
rs17467164	12	40814197	MUC19	V/I	PolyPhen = benign(0.002); SIFT = tolerated(0.96); GMAF = A: 0.0445	A/G	0.029	0.018	1.64	0.01
rs3742569	14	55818706	FBXO34	L/P	PolyPhen = benign(0.001); SIFT = tolerated(0.12); GMAF = C: 0.3926	C/T	0.379	0.441	0.77	0.008
rs1045002	14	55818517	FBXO34	I/N	PolyPhen = benign(0); SIFT = tolerated(0.51); GMAF = A: 0.3434	A/T	0.379	0.44	0.78	0.009
rs6669481	1	2.06E + 08	SLC26A9	R/G	PolyPhen = benign(0.001); GMAF = C: 0.1497	C/T	0.108	0.072	1.55	0.012
rs3742884	14	68251934	ZFYVE26	A/V	PolyPhen = benign(0.011); SIFT = tolerated(1); GMAF = A: 0.0473	A/G	0.024	0.012	2.06	0.019
rs3742883	14	68234539	ZFYVE26	N/S	PolyPhen = benign(0.002); SIFT = tolerated(0.81); GMAF = T: 0.0826	T/C	0.024	0.012	2.06	0.02
rs34094401	14	68352648	RAD51B	L/W	PolyPhen = probably_damaging(0.912); SIFT = deleterious(0.02); GMAF = G: 0.0188	G/T	0.02	0.006	3.54	0.008
rs1442138	4	90816294	MMRN1	T/A	PolyPhen = benign(0.005); SIFT = tolerated(0.98); GMAF = G: 0.0684	G/A	0.092	0.054	1.79	0.004
rs12647859	4	91230579	FAM190A	G/S	PolyPhen = benign(0.172); SIFT = tolerated(0.21); GMAF = A: 0.1754	A/G	0.138	0.101	1.43	0.035
rs3886999	20	3577062	ATRN	R/K	PolyPhen = benign(0.002); SIFT = tolerated(1); GMAF = A: 0.0225	A/G	0.026	0.045	0.57	0.047
rs41309351	20	2776527	CPXM1	R/W	PolyPhen = benign(0.011); SIFT = tolerated(0.09); GMAF = A: 0.0051	A/G	0.018	0.003	5.24	0.003
rs17782078	20	3541382	ATRN	I/T	PolyPhen = benign(0.072); SIFT = tolerated(0.28); GMAF = C: 0.0225	C/T	0.026	0.045	0.58	0.046
rs34753687	19	2833943	ZNF554	G/E	PolyPhen = probably_damaging(0.938); SIFT = tolerated(1); GMAF = A: 0.0106	A/G	0.035	0.014	2.64	0.006
rs45562539	19	1918134	SCAMP4	A/T	PolyPhen = benign(0.01); SIFT = tolerated(0.22); GMAF = A: 0.0051	A/G	0.007	0.021	0.31	0.043
rs13243961	7	23240263	NUPL2	D/N	PolyPhen = possibly_damaging(0.457); SIFT = tolerated(0.92); GMAF = A: 0.0560	A/G	0.145	0.083	1.87	0
rs1547742	1	2.33E + 08	SIPA1L2	S/L	PolyPhen = benign(0.109); SIFT = tolerated(0.05); GMAF = A: 0.0680	A/G	0.055	0.1	0.52	0.001

## References

[b1] Lees A. J., Hardy J. & Revesz T. Parkinson’s disease. Lancet 373, 2055–2066, doi: 10.1016/S0140-6736(09)60492-X (2009). 19524782

[b2] Verstraeten A., Theuns J. & Van Broeckhoven C. Progress in unraveling the genetic etiology of Parkinson disease in a genomic era. Trends Genet 31, 140–149, doi: 10.1016/j.tig.2015.01.004 (2015). 25703649

[b3] Nalls M. A. . Large-scale meta-analysis of genome-wide association data identifies six new risk loci for Parkinson’s disease. Nat Genet 46, 989–993, doi: 10.1038/ng.3043 (2014). 25064009 PMC4146673

[b4] Finucane H. K. . Partitioning heritability by functional annotation using genome-wide association summary statistics. Nat Genet 47, 1228–1235, doi: 10.1038/ng.3404 (2015). 26414678 PMC4626285

[b5] Kiezun A. . Exome sequencing and the genetic basis of complex traits. Nat Genet 44, 623–630, doi: 10.1038/ng.2303 (2012). 22641211 PMC3727622

[b6] Adzhubei I. A. . A method and server for predicting damaging missense mutations. Nat Methods 7, 248–249, doi: 10.1038/nmeth0410-248 (2010). 20354512 PMC2855889

[b7] Purcell S. . PLINK: a tool set for whole-genome association and population-based linkage analyses. Am J Hum Genet 81, 559–575, doi: 10.1086/519795 (2007). 17701901 PMC1950838

[b8] Wu M. C. . Rare-variant association testing for sequencing data with the sequence kernel association test. Am J Hum Genet 89, 82–93, doi: 10.1016/j.ajhg.2011.05.029 (2011). 21737059 PMC3135811

[b9] Hernandez D. G., Reed X. & Singleton A. B. Genetics in Parkinson disease: Mendelian versus non-Mendelian inheritance. J Neurochem, doi: 10.1111/jnc.13593 (2016). PMC5155439 27090875

[b10] Franz E. A. . Congenital mirror movements: phenotypes associated with DCC and RAD51 mutations. J Neurol Sci 351, 140–145, doi: 10.1016/j.jns.2015.03.006 (2015). 25813273

[b11] Gallea C. . RAD51 deficiency disrupts the corticospinal lateralization of motor control. Brain 136, 3333–3346, doi: 10.1093/brain/awt258 (2013). 24056534

[b12] Lawton M. . Parkinson’s Disease Subtypes in the Oxford Parkinson Disease Centre (OPDC) Discovery Cohort. J Parkinsons Dis 5, 269–279, doi: 10.3233/JPD-140523 (2015). 26405788 PMC4923737

[b13] Lek M. . Analysis of protein-coding genetic variation in 60,706 humans. Nature 536, 285–291, doi: 10.1038/nature19057 (2016). 27535533 PMC5018207

[b14] Ashburner M. . Gene ontology: tool for the unification of biology. The Gene Ontology Consortium. Nat Genet 25, 25–29, doi: 10.1038/75556 (2000). 10802651 PMC3037419

[b15] Lorenzl S., Albers D. S., Narr S., Chirichigno J. & Beal M. F. Expression of MMP-2, MMP-9, and MMP-1 and their endogenous counterregulators TIMP-1 and TIMP-2 in postmortem brain tissue of Parkinson’s disease. Exp Neurol 178, 13–20 (2002). 12460604 10.1006/exnr.2002.8019

[b16] He X. . Association studies of MMP-9 in Parkinson’s disease and amyotrophic lateral sclerosis. PLoS One 8, e73777, doi: 10.1371/journal.pone.0073777 (2013). 24040066 PMC3767588

[b17] Consortium U. K. . The UK10K project identifies rare variants in health and disease. Nature 526, 82–90, doi: 10.1038/nature14962 (2015). 26367797 PMC4773891

[b18] Wirdefeldt K., Adami H. O., Cole P., Trichopoulos D. & Mandel J. Epidemiology and etiology of Parkinson’s disease: a review of the evidence. Eur J Epidemiol 26 Suppl 1, S1–58, doi: 10.1007/s10654-011-9581-6 (2011). 21626386

[b19] Wirdefeldt K., Gatz M., Reynolds C. A., Prescott C. A. & Pedersen N. L. Heritability of Parkinson disease in Swedish twins: a longitudinal study. Neurobiol Aging 32, 1923 e1921-1928, doi: 10.1016/j.neurobiolaging.2011.02.017 (2011). PMC4452894 21482443

[b20] Healy D. G. . Phenotype, genotype, and worldwide genetic penetrance of LRRK2-associated Parkinson’s disease: a case-control study. Lancet Neurol 7, 583–590, doi: 10.1016/S1474-4422(08)70117-0 (2008). 18539534 PMC2832754

[b21] Ladouceur M., Dastani Z., Aulchenko Y. S., Greenwood C. M. & Richards J. B. The empirical power of rare variant association methods: results from sanger sequencing in 1,998 individuals. PLoS Genet 8, e1002496, doi: 10.1371/journal.pgen.1002496 (2012). 22319458 PMC3271058

[b22] Foo J. N., Liu J. J. & Tan E. K. Whole-genome and whole-exome sequencing in neurological diseases. Nat Rev Neurol 8, 508–517, doi: 10.1038/nrneurol.2012.148 (2012). 22847385

[b23] Cox B. C., Cincotta M. & Espay A. J. Mirror movements in movement disorders: a review. Tremor Other Hyperkinet Mov (N Y) 2 (2012). 10.7916/D8VQ31DZ PMC3569961 23440079

[b24] Hughes A. J., Daniel S. E., Kilford L. & Lees A. J. Accuracy of the clinical diagnosis of idiopathic Parkinson’s disease: a clinico-pathological study of 100 cases. J Neurol Neurosurg Psychiatry 55, 181–184 (1992). 1564476 10.1136/jnnp.55.3.181 PMC1014720

[b25] Szewczyk-Krolikowski K. . The influence of age and gender on motor and non-motor features of early Parkinson’s disease: initial findings from the Oxford Parkinson Disease Center (OPDC) discovery cohort. Parkinsonism Relat Disord 20, 99–105, doi: 10.1016/j.parkreldis.2013.09.025 (2014). 24183678

[b26] Genomes Project C. . An integrated map of genetic variation from 1,092 human genomes. Nature 491, 56–65, doi: 10.1038/nature11632 (2012). 23128226 PMC3498066

[b27] McKenna A. . The Genome Analysis Toolkit: a MapReduce framework for analyzing next-generation DNA sequencing data. Genome Res 20, 1297–1303, doi: 10.1101/gr.107524.110 (2010). 20644199 PMC2928508

[b28] Sherry S. T. . dbSNP: the NCBI database of genetic variation. Nucleic Acids Res 29, 308–311 (2001). 11125122 10.1093/nar/29.1.308 PMC29783

[b29] Li H. . The Sequence Alignment/Map format and SAMtools. Bioinformatics 25, 2078–2079, doi: 10.1093/bioinformatics/btp352 (2009). 19505943 PMC2723002

[b30] Li H. A statistical framework for SNP calling, mutation discovery, association mapping and population genetical parameter estimation from sequencing data. Bioinformatics 27, 2987–2993, doi: 10.1093/bioinformatics/btr509 (2011). 21903627 PMC3198575

[b31] DePristo M. A. . A framework for variation discovery and genotyping using next-generation DNA sequencing data. Nat Genet 43, 491–498, doi: 10.1038/ng.806 (2011). 21478889 PMC3083463

[b32] McLaren W. . Deriving the consequences of genomic variants with the Ensembl API and SNP Effect Predictor. Bioinformatics 26, 2069–2070, doi: 10.1093/bioinformatics/btq330 (2010). 20562413 PMC2916720

[b33] Danecek P. . The variant call format and VCFtools. Bioinformatics 27, 2156–2158, doi: 10.1093/bioinformatics/btr330 (2011). 21653522 PMC3137218

[b34] Lill C. M. . Comprehensive research synopsis and systematic meta-analyses in Parkinson’s disease genetics: The PDGene database. PLoS Genet 8, e1002548, doi: 10.1371/journal.pgen.1002548 (2012). 22438815 PMC3305333

