# Supplementary material for: Whole-exome sequencing of 228 patients with sporadic Parkinson’s disease
Source: Sci Rep. 2017 Jan 24;7:41188. doi: 10.1038/srep41188 (PMC5259721; doi:10.1038/srep41188)
Supplement: Supplementary Information [file srep41188-s1.pdf]

Supplementary data for:

"Whole-exome sequencing of 228 patients with sporadic Parkinson's disease"

Cynthia Sandor[1,3,+], Frantisek Honti[3,6+], Wilfried Haerty[3,7], Konrad Szewczyk-Krolikowski[1,4], Paul Tomlinson[1,4], Sam Evetts[1,4], Stephanie Millin[1,3], Thomas Keane[5], Shane McCarthy[5], Richard Durbin[5], Kevin Talbot[1,4], Michele Hu[1,4], Caleb Webber[1,3*], Chris P. Ponting[1,3,5,8*] and Richard Wade-Martins[1,2,*]

[1]Oxford Parkinson's Disease Centre, University of Oxford, Oxford, United Kingdom

[2]Department of Physiology, Anatomy and Genetics, University of Oxford, Oxford, OX1 3QX, United Kingdom

[3]MRC Functional Genomics Unit, Department of Physiology, Anatomy and Genetics, University of Oxford, Oxford, OX1 3PT, United Kingdom

[4]Nuffield Department of Clinical Neurosciences, John Radcliffe Hospital, West Wing Level 6, Headley Way, Oxford OX3 9DU, United Kingdom

[5]Wellcome Trust Sanger Institute, Wellcome Trust Genome Campus, Hinxton, Cambridge, CB10 1SA, United Kingdom

[6]Current address: Royal Brompton & Harefield NHS Foundation Trust, Sydney Street, London SW3 6NP United Kingdom

[7]Current address: Earlham Institute Norwich Research Park Innovation Centre, Colney Lane, Norwich NR4 7UH

[8]Current address: MRC Human Genetics Unit, MRC IGMM, University of Edinburgh, Edinburgh, United Kingdom

*To whom correspondence should be addressed:

richard.wade-martins@dpag.ox.ac.uk or chris.ponting@igmm.ed.ac.uk or michele.hu@ndcn.ox.ac.uk or caleb.webber@dpag.ox.ac.uk

+These authors contributed equally to this work

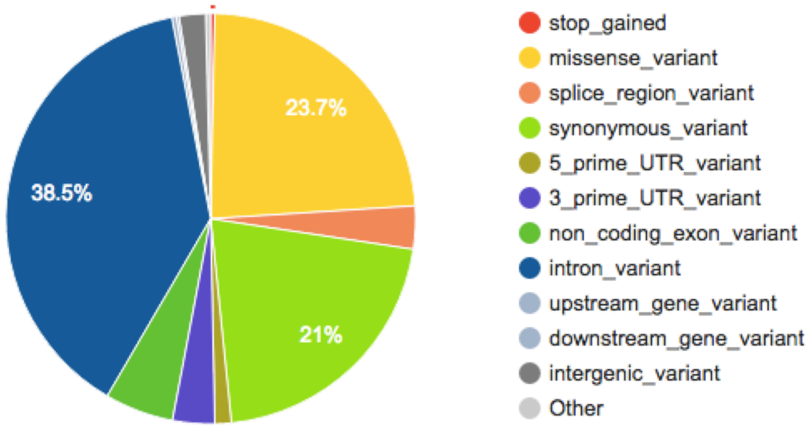

Supplementary Figure1: Proportion in term of most severe consequences of 94369 SNV.

Variant consequence annotations were added using the Ensembl Variant Effect Predictor

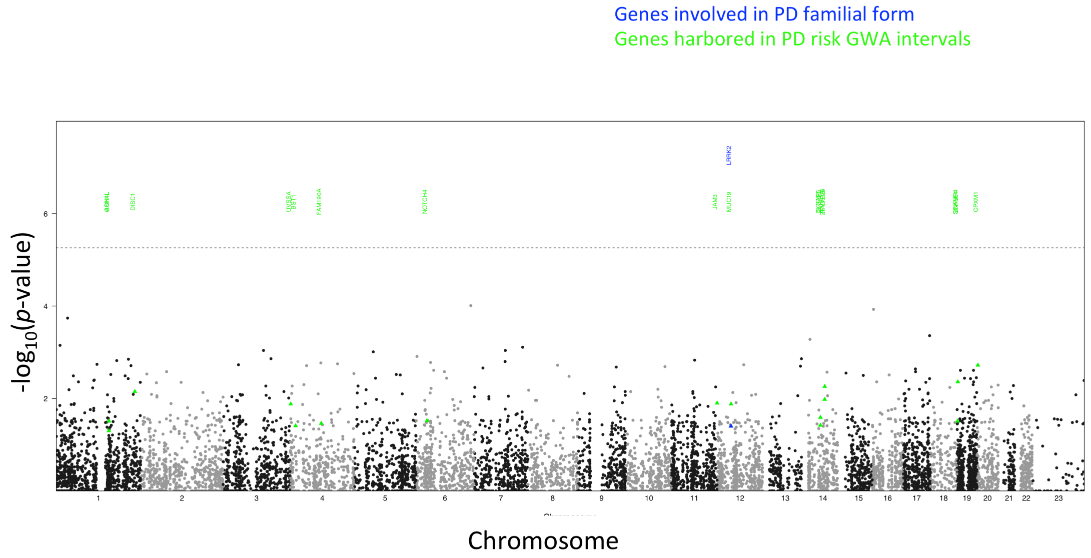

Supplementary Figure 2: Manhattan plot showing the significance (-log$_{10}$(p-value)) of association SKAT test association of x genes holding y non-synomous mutations

The horizontal line indicates the Bonferroni multiple testing correction threshold at p = $2.4x10^{-6}$. The blue and green points represent the significance of association for non- synonymous variants within genes involved in PD familial form (mono) or harboured in one of 26 published PD GWA intervals (GWA) respectively. The names of genes are given in the top part of plot in blue and green for mono and GWA genes respectively. For 26 GWA intervals, only the name of gene including the most significant association is showed.

Supplementary Table1: List of genes involved in PD familial form

Gene Symbol
PARK2
PINK1
PARK7
NEDD4
GBA
SNCA
MAPT
LRRK2
GCH1
RAB7L1
BST1
MCCC1
STK39
ACMSD
TMEM163
TMEM175
GAK
DGKQ
DNAJC13
DCTN1
VPS35
ATP13A2
PLA2G6
FBXO7
SYNJ1
DNAJC6
CHCHD2

Supplementary Table2: Gene Ontologies annotations enrichment analyses for 300 genes with most PD associated Parkinson NS SNV

Pathway	*p*-value (Nominal)	*p*-value (FDR adjusted)	# genes expected	# genes observed
extracellular matrix part(GO:0044420)	3.80E-05	0.02	6	11
extracellular matrix disassembly(GO:0022617)	5.04E-05	0.02	5	9
